# Development and external validation of a prediction model for the risk of relapse in psoriasis after discontinuation of biologics

**DOI:** 10.3389/fmed.2024.1488096

**Published:** 2024-11-26

**Authors:** Shan Huang, Bailin Chen, Yiming Qi, Xingwu Duan, Yanping Bai

**Affiliations:** ^1^Dongzhimen Hospital, Beijing University of Chinese Medicine, Beijing, China; ^2^Graduate School, Beijing University of Chinese Medicine, Beijing, China; ^3^Department of Dermatology, China-Japan Friendship Hospital, National Center for Integrative Chinese and Western Medicine, Beijing, China

**Keywords:** psoriasis, biologics, prediction model, risk factor, relapse

## Abstract

**Background:**

Some patients with psoriasis experience relapses shortly after discontinuation of biologics. However, there is a lack of risk prediction tools to identify those at high risk of relapse.

**Objective:**

To develop and validate a risk prediction model for psoriasis relapse after biologics discontinuation.

**Methods:**

Publications from PubMed, EMBASE, Medline, and the Cochrane Library were systematically searched and meta-analyses were conducted to identify risk factors for psoriasis relapse after biologics discontinuation. Statistically significant risk factors were identified and used to create a risk assessment model weighted by the impact of each factor. The model was externally validated using a cohort of 416 Chinese psoriasis patients.

**Results:**

Eight studies (*N* = 2066) were included in the meta-analysis. Body mass index (BMI), smoking, disease duration, comorbid psoriatic arthritis (PsA), remission speed and extent during treatment, history of biologic therapy, and therapy duration were identified as correlates of relapse in the meta-analysis and were incorporated into the prediction model. The median age of the 416 patients in the validation cohort was 41.5 (IQR 32, 53) years, with 63% male, and a baseline PASI score of 15.4 (IQR 10.5, 21). It was verified that the area under the curve (AUC) of the prediction model was 0.796 (95% CI, 0.753–0.839), with an optimal cut-off value of 11.25 points, sensitivity of 65.1%, and specificity of 82.2%.

**Conclusion:**

Multivariate models using available clinical parameters can predict relapse risk in psoriasis patients after biologics discontinuation. Early individual identification of patients at risk of relapse, and screening of candidate cohorts for long-term treatment or dose reduction may benefit both patients and physicians.

## 1 Introduction

Psoriasis, an immune-mediated chronic systemic disease, affects approximately 2–3% of the global population, imposing a substantial disease burden and negatively impacting quality of life and systemic health (1, 2). The advent of biologics has transformed psoriasis treatment, leading to short-term reductions or clearances and effective control over inflammation and disease activity (3, 4). However, relapse post-treatment discontinuation poses a considerable therapeutic challenge, despite significant advances in treatment through the widespread use of biologics (5, 6).

Previous studies have shown that more than half of patients relapse within 6 months of discontinuation, despite achieving remission or even complete clearance with treatment (7). Psoriasis relapse has been linked to localized residual inflammation and disease memory. Research has demonstrated that genes encoding inflammatory factors and skin structural proteins in visually resolved lesions differ from those in healthy skin (8). Furthermore, tissue-resident memory T cells in these resolved lesions continue to persist, retaining their ability to produce inflammatory factors associated with psoriasis relapse, such as interleukin-22 and interleukin-17A (8, 9). The etiology and pathogenesis of relapse are not fully understood, and there is debate regarding the associated risk factors (10). For instance, while Warren et al. reported that higher body mass index (BMI) promotes psoriasis relapse (11), Topaloğlu Demir et al. (12) found no association between BMI and relapse. These conflicting findings complicate the effective clinical management of relapse.

The prediction model serves as a tool for predicting future trends, behaviors, or outcomes based on historical data or established information. It utilizes various mathematical and statistical techniques to analyze and learn from patterns within the data (13, 14). In recent years, medical prediction models have garnered increasing attention due to their substantial value in disease diagnosis and prognosis. However, there remains a deficiency of clinically useful models specifically designed for predicting psoriasis relapse.

Therefore, we conducted a meta-analysis to identify risk factors for psoriasis relapse following the discontinuation of biologics and developed a practical individual prediction model for relapse based on factors routinely available in clinical practice. Moreover, we established an external cohort to validate the model’s performance. This research aims to identify individuals at high risk of relapse, enabling early personalized interventions to reduce relapse rates and enhance long-term disease prognosis. Additionally, it may assist in identifying patients at low risk of relapse who could benefit from dose reduction, potentially alleviating the economic burden on the healthcare system.

## 2 Methods

### 2.1 Meta-analysis

The meta-analysis was conducted and reported following the Preferred Reporting Items for Systematic Reviews and Meta-Analyses (PRISMA) statement (15). The study protocol was registered with the International Registry for Prospective Systematic Evaluation (PROSPERO) and the registration number is CRD 42024543820.

#### 2.1.1 Search strategy

We searched electronic databases, including Pubmed, EMBASE, Medline, and the Cochrane Library, for relevant literature from the inception of database to May 1, 2024. The search formula was formulated according to the PICO criteria (Supplementary Table S1). After duplicates were removed, initial screening of the publications was conducted by assessing the titles and abstracts, followed by a full-text review to identify the final articles (Supplementary Figure S1). The literature search and screening was carried out independently by two researchers (Huang Shan and Chen Bailin), and disagreements were resolved through discussion.

#### 2.1.2 Article selection

Articles were included in this study if they met the following criteria: (1) the study population consisted of patients with plaque psoriasis; (2) the therapeutic agent was a biologic; (3) patients achieved clinical remission during treatment with the biologic; (4) follow-up was at least 6 months after biologic discontinuation; (5) risk factors for relapse were reported, extractable odds ratios (ORs), relative risks (RRs), or hazard ratios (HRs), and 95% confidence intervals (Cls); and (6) the full text was available. The following studies were excluded: (1) conference abstracts, reviews, commentaries, author responses, meta-analyses, and case reports; (2) unclear definitions of remission or relapse; and (3) study populations with fewer than 30 patients. If multiple studies describing the same outcome in the same population were available, only the most complete one was included.

#### 2.1.3 Data extraction and quality assessment

Data extraction and quality assessment were conducted independently by two authors (Huang Shan and Chen Bailin), and disagreements were resolved through discussion. If a study reported multiple effect values for the same factor, we selected only the estimate with the most adjusted variables and the longest follow-up time. The extracted data included authors, publication year, sample size, interventions, risk factors, effect sizes, and 95% CIs for the risk factors. The quality of the studies was evaluated using the Newcastle-Ottawa Scale (16), which was performed independently by the two authors.

### 2.2 Validation cohort

We reported the validation of the prediction model following the Transparent Reporting of Multivariate Predictive Models for Individual Prognosis or Diagnosis (TRIPOD) statement (17). The study received approval from the ethics committees of China-Japan Friendship Hospital (2019-159-K108) and Dongzhimen Hospital (DZMEC-KY-2019-164). Written informed consent was obtained from each patient.

#### 2.2.1 Study population

Patients with moderate-to-severe psoriasis who discontinued treatment after achieving remission with biologics between October 2019 and April 2023 at China-Japan Friendship Hospital and Dongzhimen Hospital were identified. Remission was defined as a 75% reduction in the Psoriasis Area and Severity Index (PASI) from baseline (achievement of PASI 75). Patients’ baseline information and PASI scores during treatment were extracted from the Psoriasis Case Registry System (ClinicalTrials.gov identifier: ChiCTR1900021629) or electronic medical records.

#### 2.2.2 Endpoint event

The endpoint event of the study was relapse, defined as the deterioration of skin lesions post-discontinuation in patients who had met remission criteria, with an increase in PASI score to more than 25% of the baseline (loss of PASI 75) or the resumption of systemic therapy or phototherapy. During the 6-month follow-up, physicians recorded patients’ PASI scores and treatments through offline clinics, Internet health services, or video calls to assess for relapse.

### 2.3 Statistical analysis

We reported the validation of the prediction model following the Transparent Reporting of Multivariate Predictive Models Used for Individual Prognosis or Diagnosis (TRIPOD) statement (17). The study received approval from the ethics committees of China-Japan Friendship Hospital (2019-159-K108) and Dongzhimen Hospital (DZMEC-KY-2019-164). Written informed consent was obtained from each patient.

#### 2.3.1 Meta-analysis

The extracted effect values were converted into RR values, and the detailed extraction and conversion transformation strategy is described in Supplementary Tables S2, S3. RR values and 95% CIs for each risk factor were pooled separately using the corresponding meta-analysis model selected based on the heterogeneity of the studies. Cochran’s *Q* test and *I*^2^ statistics were utilized to test statistical heterogeneity between studies. If there was statistically significant heterogeneity (*p-*value <0.10 or *I*^2^ > 50%), the pooled RRs and 95% CIs were calculated using a random-effects model; otherwise, they were generated by a fixed-effects model. Sensitivity analyses were conducted by altering the effects model to assess the robustness of the results.

#### 2.3.2 Model development

First, we examined the statistically significant risk factors from the meta-analyses mentioned above and identified the factors included in the model after removing overlapping variables with covariates. Second, we calculated the *β* coefficients of all risk factors in the model using the pooled RR values, which represent the multiplicative increase in an individual’s risk of developing a particular disease for each unit increase in the risk variable (18).


$$\beta =\ln\;\left(RR\right)$$

The *β* coefficients were then multiplied by 10 to obtain the corresponding score for each risk factor. Finally, all risk factors were stratified and evaluated to develop a risk prediction model for psoriasis relapse. The total score was calculated by summing the scores for each risk factor. For individuals, a higher cumulative score indicates a greater risk of relapse after discontinuing medication.

#### 2.3.3 Model validation

The receiver operating characteristic (ROC) curve was plotted based on the total scores from the prediction model, and decision curve analysis (DCA) was performed. The sensitivity, specificity, and area under the curve (AUC) value of the model were calculated. The AUC, indicative of predictive accuracy, ranges from 0.5 to 1.0, with higher values denoting greater accuracy. The optimal cut-off point with high sensitivity and specificity was identified based on the Youden index. Patients were divided into four groups according to the optimal cut-off and interquartile range: low risk, intermediate risk, high risk, and very high risk. Kaplan–Meier curve analysis was used to assess the cumulative risk of relapse in the different groups.

Statistical analyses were performed using R version 4.3.3 and Stata 12.0 (Stata Corp, College Station, TX). All tests were considered statistically significant with a two-sided *p*-value of <0.05, except the heterogeneity test with a *p*-value of <0.1.

## 3 Results

### 3.1 Meta-analysis

#### 3.1.1 Characteristics of included studies

A total of 3,769 articles were retrieved from the database, and eight studies (four cohort studies and four case–control studies involving 2,066 patients) were included based on the inclusion and exclusion criteria (Table 1 and Supplementary Table S4). According to the Newcastle-Ottawa Scale (NOS), the scores of the included studies ranged from 5 to 7, with 7/8 being of high quality. These studies identified 15 risk factors for relapse, including age, gender, body mass index, body weight, alcohol consumption, smoking, baseline PASI, baseline BSA, disease duration (years), disease duration (>2 years), psoriatic arthritis (PsA), speed of lesion remission, extent of lesion remission, history of treatment with biologics, and course of treatment.

**Table 1:** Characteristics of the included studies.

Study	Type of study	Region	Publication year	Eligible participants	Biologics for treatment	Definition of remission	Definition of relapse	Newcastle-Ottawa score
Lebwohl et al. (2023) (35)	Cohort study	Worldwide	2023	220	SEC	Achievement of PASI 75	Loss of PASI 50	7
Topaloğlu Demir. et al. (2023) (12)	Case–control study	Turkey	2023	169	ADA, ETA, INF, CER, UST, SEC, IXE	Achievement of PASI 75	Loss of PASI 50	6
Warren et al. (2021) (11)	Case–control study	The United Kingdom	2021	233	TIL	Achievement of PASI 75	Loss of PASI 75	6
Owczarek et al. (2021) (25)	Cohort study	Poland	2021	705	ADA, UST, ETA, INF, SEC, IXE	Achievement of PASI75 or PASI50-75 with simultaneous DLQI/CDLQI scale by at least 5 points	An increase in one of PASI, DLQI, (or CDLQI) or body surface area (BSA) by 50% as compared to their respective values calculated in the time of treatment termination and PASI being greater than 10	7
Chiu et al. (2023) (19)	Cohort study	China	2023	202 (304TEs)	UST	Achievement of PASI 50	Loss of PASI 50 or PASI ≥10 and BSA ≥10%	6
Stinco et al. (2019) (20)	Case–control study	Italy	2019	270	ADA, ETA, INF	PASI <3 for at least 12 months	Loss of PASI 50	6
Umezawa et al. (2019) (36)	Case–control study	Italy	2019	70	IXE	Achievement of PASI 75	Loss of PASI 50	5
Huang and Tsai (2019) (29)	Cohort study	China (Taiwan)	2019	95	EFA, ALE, ETA, Tofacitinib, IXE, SEC, UST, GUS	Achievement of PASI 75	PGA ≥2 or loss of PASI 75	6

#### 3.1.2 Risk factors for relapse

**Figure 1:**
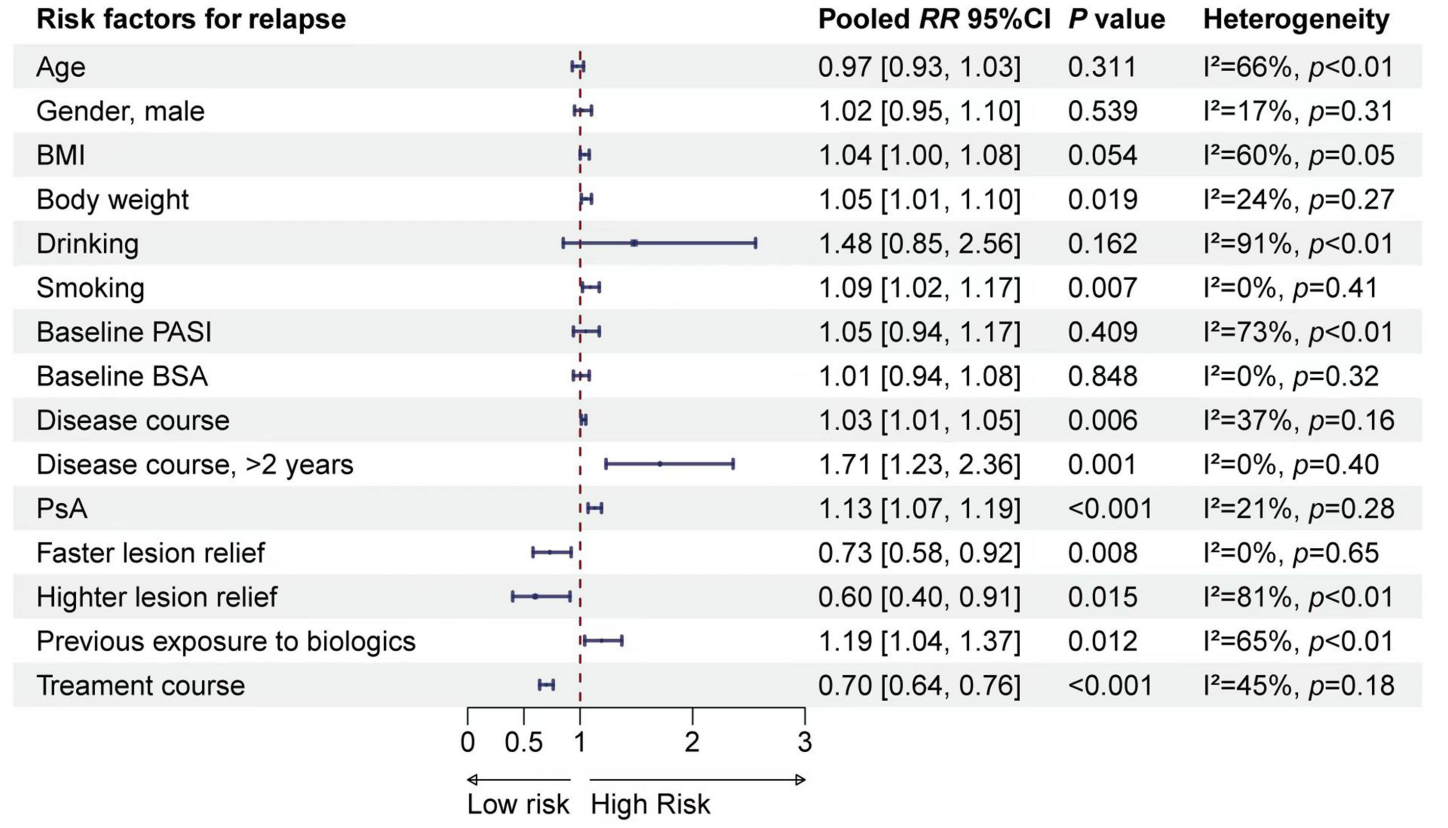
Meta-analysis of risk factors for psoriasis relapse after discontinuation of biologics.

A total of 15 risk factors were included in the meta-analysis. The results suggested that BMI, weight, smoking, disease duration (years), disease duration (>2 years), PsA, speed of lesion remission, degree of lesion remission, previous exposure to biologics, and duration of therapy were associated with relapse (Figure 1). Detailed results of the meta-analyses, heterogeneity tests, and sensitivity analyses for each risk factor are provided in Supplementary Figure S2. No publication bias test was conducted due to the limited number of studies included for a valid test.

### 3.2 Model development

The meta-analysis identified 10 risk factors associated with psoriasis relapse. Considering multicollinearity, we selected BMI instead of weight and disease duration (>2 years) instead of disease duration (years) for inclusion in the model. The final model included eight variables: BMI (RR = 1.03, 95% CI 1.00–1.07), smoking (RR = 1.09, 95% CI 1.02–1.17), disease duration >2 years (RR = 1.71, 95% CI 1.23–2.36), PsA (RR = 1.13, 95% CI 1.07–1.19), faster lesion remission (RR = 0.73, 95% CI 0.51–0.92), higher skin lesion remission (RR = 0.60, 95% CI 0.40–0.91), previous exposure to biologics (RR = 1.19, 95% CI 1.04–1.37), and duration of therapy (RR = 0.70, 95% CI 0.64–0.76). The *β* coefficients of the risk factors were calculated, and a risk prediction model for psoriasis relapse after discontinuation of biologics was developed based on the grading and scoring of the risk factors (Table 2).

**Table 2:** Risk score model for predicting psoriasis relapse after discontinuation of biologics.

Risk factors for relapse	Risk stratification	Points
BMI (kg/m^2^)	<24	0
	24 ~ 27.99	1.5
	≥28	3
Smoking	No	0
	Yes	1
Disease course (years)	≤2	0
	>2	5
PsA	No	0
	Yes	1
Higher lesion relief^a^	No	0
	Yes	5
Faster lesion relief^a^	No	0
	Yes	3
Previous exposure to biologics	No	0
	Yes	2
Treatment course (months)^b^	≤10.75	3.5
	>10.75	0

^a^Higher lesion relief was defined as achieving PASI 90 and Faster lesion relief was defined as achieving remission within 8 weeks (PASI 75). ^b^The optimal cut-off value for the course of treatment according to the Youden index was 10.75 months (Supplementary Figure S3).

### 3.3 Model validation

#### 3.3.1 Baseline characteristics of the validation cohort

The validation cohort consisted of 416 patients with psoriasis who had achieved remission with biologic therapy and then discontinued treatment. The median age of the patients was 41.5 years, and 262 (63%) were male. Within 6 months past-discontinuation, 186 (44.7%) patients experienced a relapse. Detailed characteristics of the validation cohort are presented in Table 3.

**Table 3:** Baseline characteristics of the validation cohort.

Characteristics	*N* = 416
Age, median [Q1, Q3]	41.5 (32, 53)
Gender (male), %	262 (63)
BMI (kg/m^2^)	
≤24, %	171 (41.1)
24 ~ 28, %	170 (40.9)
>28, %	75 (18)
PASI score, median [Q1, Q3]	15.4 (10.5,21)
Disease course >2 years, %	374 (89.9)
Biologics	
Adalimumab, %	120 (28.8)
Secukinumab, %	232 (55.8)
Ixekizumab, %	64 (15.4)
Treatment course (months)	10 (8,12)
Achieving PASI 75 in 8 weeks, %	305 (73.3)
Achievement of PASI 90, %	351 (84.4)
Family history, %	94 (22.6)
Relapse, %	186 (44.7)
Smoking, %	178 (42.8)
PsA, %	39 (9.4)
Previous exposure to biologics, %	72 (17.3)

#### 3.3.2 Performance of the model

**Figure 2:**
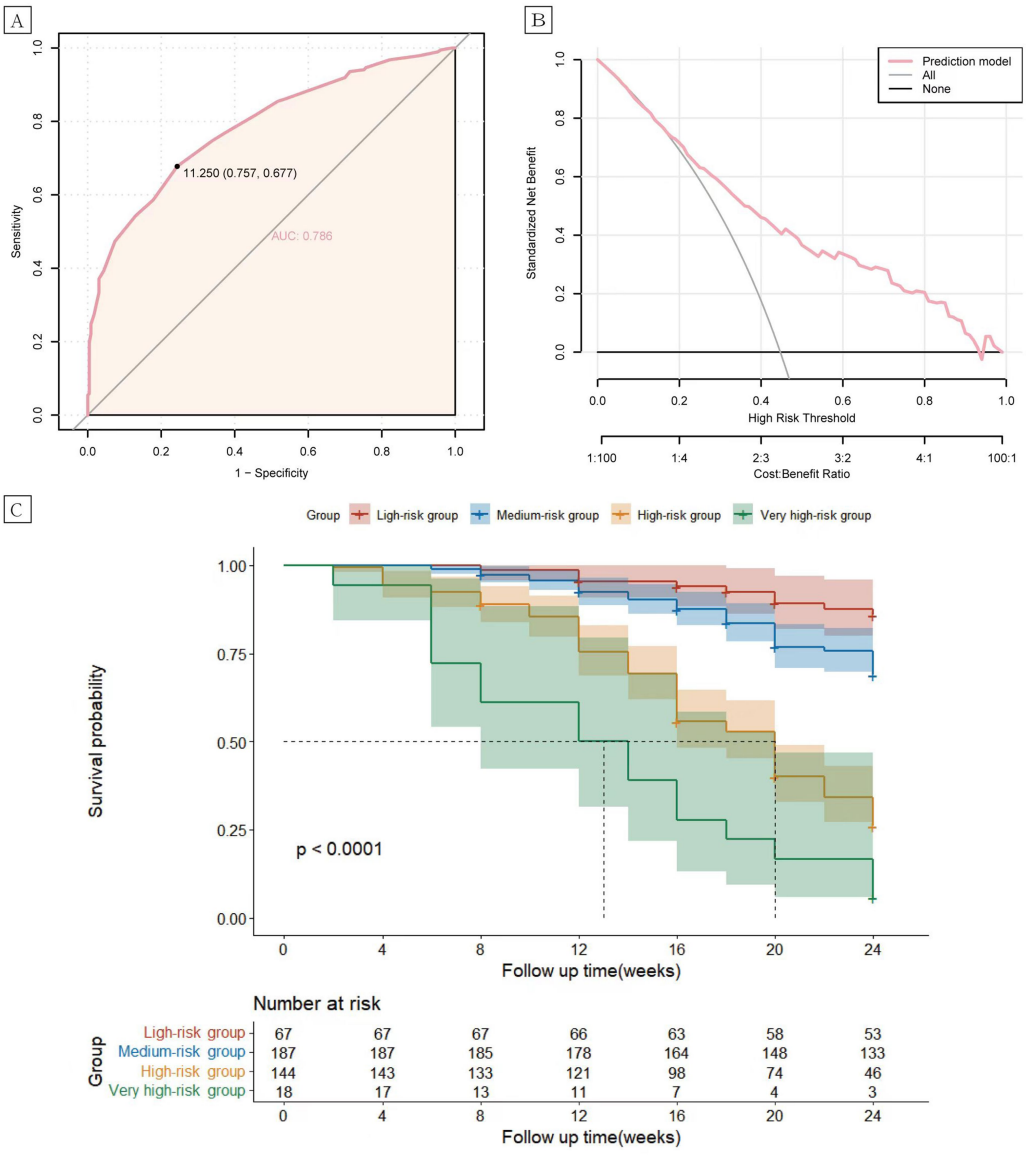
Receiver operating characteristic (ROC) curves **(A)** and clinical decision analysis (DCA) curves **(B)** for the psoriasis relapse prediction model. Kaplan–Meier curves **(C)** of the cumulative risk of relapse in patients in the low, intermediate, high, and very high-risk groups.

In the validation cohort, the AUC value of the risk prediction model was 0.796 (95% CI 0.753–0.839). The optimal cutoff value was determined to be 11.25 by the Youden index, with a sensitivity of 0.651 and a specificity of 0.822, which indicates good predictive performance of the model (Figure 2A). The 6-month relapse rate of psoriasis after discontinuation of biologics has been reported to be 25.4–59.7% in previous literature (19–21). Within this range, the decision curve lies above the none and all lines, suggesting clinical utility for the model (Figure 2B). Based on the total risk score, patients in the validation cohort were categorized into four groups: low-risk (*n* = 67, 0–6 points), intermediate-risk (*n* = 187, 6.5–11.25 points), high-risk (*n* = 144, 11.25–17.5 points), and very high-risk (*n* = 18, 18–23.5 points). Compared to the low-risk group, the intermediate-risk group (HR = 2.43, 95% CI 1.20–4.90, *p* < 0.001), the high-risk group (HR = 8.78, 95% CI 4.44–17.38, *p* < 0.001), and very high-risk group (HR = 19.54, 95% CI 8.67–44.02, *p* < 0.001) exhibited significantly elevated risk of relapse (Figure 2C).

## 4 Discussion

Psoriasis remains incurable, and due to the high relapse rate after discontinuation of therapy, biologics are recommended for long-term maintenance therapy (5, 22). However, patients in remission often stop treatment due to secondary loss of efficacy, high financial burden, and concerns about long-term therapy safety (23, 24). The differences in patient outcomes after discontinuation of biologics are significant. More than half of these patients experience relapse within 6 months, while some enjoy prolonged periods of remission (7, 25). To date, no sophisticated models have been developed to predict the risk of relapse after discontinuation of biologics. Gordon et al. (26) discovered that maintenance of the PASI90 response after discontinuation of Guselkumab was associated with sustained suppression of interleukin (IL)-17A, IL-17F, and IL-22. However, cytokine levels were not predictive of psoriasis relapse, as their increase lagged behind the rise in PASI scores. Rivera et al. (27) constructed a multivariate linear regression model for psoriasis relapse, but the performance and clinical decision-making ability of the model have not been validated. Additionally, the small sample size used to develop the model may introduce potential biases.

Our study utilized meta-analysis based on eight published moderate-to-high quality clinical studies to identify 10 risk factors influencing psoriasis relapse. These include BMI, body weight, smoking, disease duration (years), disease duration (>2 years), psoriatic arthritis, rate of skin lesion resolution, extent of skin lesion resolution, history of biologic therapy, and treatment duration. The inclusion of both BMI and body weight in the predictive model could lead to overfitting and diminish the model’s performance due to their correlation. BMI was selected over body weight as it more accurately reflects an individual’s physique and metabolic state, and evidence supports its stronger association with the efficacy of biologic therapy for psoriasis compared to body weight (28). Disease duration was modeled as a binary variable (≤2 years or >2 years) to simplify the model and align with the current consensus in psoriasis relapse research that a 2-year threshold is significant for distinguishing disease chronicity (25, 29–31). In the end, 8 risk factors were incorporated into a prediction model designed to forecast the risk of psoriasis relapse after discontinuation of biologics therapy, with each factor weighted by its impact.

The prediction model exhibited robust performance (AUC = 0.796) and clinical decision-making ability, capability within the validation cohort, suggesting its practical utility in clinical settings. Furthermore, all variables integrated into the prediction model are clinically accessible non-invasively, highlighting the model ‘s practicality and ease of use. Moreover, the prediction model demonstrated excellent clinical discrimination, with significant differences in relapse rates observed among patients classified into low-risk, intermediate-risk, high-risk, and very high-risk groups based on their total scores in the validation cohort (*p* < 0.001).

Translating predictive outcomes into clinical decisions is imperative, particularly when guided by a comprehensive and precise stratification of relapse risk. For patients classified as high-risk and very high-risk with scores above 17.5, long-term maintenance therapy using biologics is advised, with a minimum treatment duration of 10.75 months as informed by ROC analysis. Treatments shorter in duration may not be effective in managing the inflammatory environment, even with lesion resolution. Notably, more than half of the risk factors incorporated in this model are modifiable, suggesting that weight reduction, smoking cessation, timely treatment initiation, and extended therapy duration may mitigate the incidence of psoriasis relapse following biologic discontinuation and enhance long-term prognosis.

Furthermore, it is also clinically important to identify low-risk relapse populations using a predictive model. Dose reductions are common in long-term treatment of psoriasis, yet there is ambiguity regarding the identification of individuals suitable for such reductions, and indiscriminate reductions potentially leading to premature relapses (32–34). Patients with a risk score of ≤6, identified as low risk, may be candidates for dose reduction, with the recommendation that this occurs post 10.75 months of treatment. For those with low relapse risk and cautionary factors for biologics, such as chronic infections, abnormal liver function, or a history of tumors, dose reduction or discontinuation may be considered once skin lesion control is achieved. Individualized determinations based on the prediction model can alleviate the healthcare burden, mitigate the adverse effects of long-term medication, and protect patient prognosis. By diminishing medical interventions, patients are afforded the opportunity to live without the ongoing necessity of treatment, aligning their lifestyle with that of healthy individuals, and consequently improving their quality of life.

## 5 Limitations

Firstly, heterogeneity between studies was unavoidable due to variations in study design, geographic region, and patient baseline characteristics, potentially impacting the reliability of the meta-analysis results. Secondly, the limited number of studies included for certain risk factors, such as alcohol consumption and baseline BSA, may have biased the results. Thirdly, the candidate variables identified through secondary data analyses did not include potential biomarkers and failed to account all potential risk factors associated with psoriasis relapse. Fourthly, while the meta-analysis included participants from various countries and regions, the validation cohort was composed solely of Chinese patients, which may affect the generalizability of the findings. Therefore, further validation of the model’s predictive performance using multicenter external cohorts is necessary.

## 6 Conclusion

This study found that BMI, weight, smoking, disease duration, PsA, the speed and degree of lesion remission during treatment, history of biologic therapy, and therapy duration were associated with psoriasis relapse following biologic discontinuation. A multivariate model leveraging routinely collected clinical data was developed to predict the relapse risk of psoriasis vulgaris post-biologic withdrawal, potentially aiding in individualized, long-term management of psoriasis. However, additional validation of the model’s clinical utility is necessary.

## Data Availability

The original contributions presented in the study are included in the article/Supplementary material, further inquiries can be directed to the corresponding authors.
