# Supplementary material for: Development and external validation of a prediction model for the risk of relapse in psoriasis after discontinuation of biologics
Source: Front Med (Lausanne). 2024 Nov 26;11:1488096. doi: 10.3389/fmed.2024.1488096 (PMC11628277; doi:10.3389/fmed.2024.1488096)
Supplement: Supplementary file 1 [file Table_1.DOCX]

Supplementary Material

# Supplementary Figures and Tables

eTable 1. Search strategy and results

eTable 2. Effect value conversion strategy

eTable 3. Effect value conversion strategy

eFigure 1. Flowchart of literature search and screening

eFigure 2. Meta-analysis, heterogeneity test and sensitivity analysis of risk factors for psoriasis relapse after discontinuation of biologics. (a) Age, (b)gender (male), (c) BMI, (d) body weight, (e) drinking, (f) smoking, (g) baseline PASI, (h) baseline BSA, (i) disease course (years), (j) disease course (≥2 years), (k) PsA, (l) faster lesion relief, (m) higher lesion relief, (n) previous exposure to biologics, (o) treatment course

eTable 4. Demographic information of the studies included in the meta-analysis

eFigure 3. ROC curve analysis of treatment duration to predict psoriasis relapse

eTable 1. Search strategy and results

| **Database** | **Search Strategy** | **Result** |
| --- | --- | --- |
| Pubmed | (psoriasis[MeSH Terms]) OR (Psoriases[Title/Abstract]) OR (plaque psoriasis[Title/Abstract]))  ((Recurrence[MeSH Terms]) OR (drug tapering[MeSH Terms]) OR (Recurrences[Title/Abstract]) OR (Recrudescence[Title/Abstract]) OR (Recrudescences[Title/Abstract]) OR (Relapse[Title/Abstract]) OR (Relapses[Title/Abstract]) OR (interruption[Title/Abstract]) OR (stopped[Title/Abstract]) OR (stop[Title/Abstract]) OR (discontinuation[Title/Abstract]) OR (discontinue[Title/Abstract]) OR (withdrawn[Title/Abstract]) OR (withdrawal[Title/Abstract])  (Biological Products[MeSH Terms]) OR (biologics[Title/Abstract]) OR (biologicals[Title/Abstract]) OR (biological agent[Title/Abstract]) OR (targeted therapies[Title/Abstract]) OR (tnf inhibitor*[Title/Abstract]) OR (antitumor necrosis factor[Title/Abstract]) OR (anti tumour necrosis factor[Title/Abstract]) OR (IL-12/23 inhibitor*[Title/Abstract]) OR (IL-17 inhibitor*[Title/Abstract]) OR (IL-23 inhibitor*[Title/Abstract]) OR (interleukin-12/23 inhibitor*[Title/Abstract]) OR (interleukin-17 inhibitor*[Title/Abstract]) OR (interleukin-23 inhibitor*[Title/Abstract]) OR (Abatacept[Title/Abstract]) OR (Adalimumab[Title/Abstract]) OR (Bimekizumab[Title/Abstract]) OR (Brodalumab[Title/Abstract]) OR (Certolizumab[Title/Abstract]) OR (Certolizumab Pegol[Title/Abstract]) OR (Deucravacitinib[Title/Abstract]) OR (Etanercept[Title/Abstract]) OR (Golimumab[Title/Abstract]) OR (Guselkumab[Title/Abstract]) OR (Infliximab[Title/Abstract]) OR (Ixekizumab[Title/Abstract]) OR (Netakimab[Title/Abstract]) OR (Risankizumab[Title/Abstract]) OR (Secukinumab[Title/Abstract]) OR (Sonelokimab[Title/Abstract]) OR (Tildrakizumab[Title/Abstract]) OR (Ustekinumab[Title/Abstract])  #1 AND #2 AND #3 | 958 |
| Medline | MH=(psoriasis)  TI=(Psoriases) OR AB=(Psoriases) OR TI=(plaque psoriasis) OR AB=(plaque psoriasis)  #1 OR #2  MH=(Recurrence) OR MH=(drug tapering)  TI=(Recurrences) OR AB=(Recurrences) OR TI=(Recrudescence) OR AB=(Recrudescence) OR TI=(Recrudescences) OR AB=(Recrudescences) OR TI=(Relapse) OR AB=(Relapse) OR TI=(Relapses) OR AB=(Relapses) OR TI=(interruption) OR AB=(interruption) OR TI=(stopped) OR AB=(stopped) OR TI=(stop) OR AB=(stop) OR TI=(discontinuation) OR AB=(discontinuation) OR TI=(discontinue) OR AB=(discontinue) OR TI=(withdrawn) OR AB=(withdrawn) OR TI=(withdrawal) OR AB=(withdrawal)  #4 OR #5  MH=(Biological Products)  TI=(biologics) OR AB=(biologics) OR TI=(biologicals) OR AB=(biologicals) OR TI=(biological agent) OR AB=(biological agent) OR TI=(targeted therapies) OR AB=(targeted therapies) OR TI=(tnf inhibitor*) OR AB=(tnf inhibitor*) OR TI=(antitumor necrosis factor) OR AB=(antitumor necrosis factor) OR TI=(anti tumour necrosis factor) OR AB=(anti tumour necrosis factor) OR TI=(IL-12/23 inhibitor*) OR AB=(IL-12/23 inhibitor*) OR TI=(IL-17 inhibitor*) OR AB=(IL-17 inhibitor*) OR TI=(IL-23 inhibitor*) OR AB=(IL-23 inhibitor*) OR TI=(Abatacept) OR AB=(Abatacept) OR TI=(Adalimumab) OR AB=(Adalimumab) OR TI=(Bimekizumab) OR AB=(Bimekizumab) OR TI=(Brodalumab) OR AB=(Brodalumab) OR TI=(Certolizumab) OR AB=(Certolizumab) OR TI=(Certolizumab Pegol) OR AB=(Certolizumab Pegol) OR TI=(Deucravacitinib) OR AB=(Deucravacitinib) OR TI=(Etanercept) OR AB=(Etanercept) OR TI=(Golimumab) OR AB=(Golimumab) OR TI=(Guselkumab) OR AB=(Guselkumab) OR TI=(Infliximab) OR AB=(Infliximab) OR TI=(Ixekizumab) OR AB=(Ixekizumab) OR TI=(Netakimab) OR AB=(Netakimab) OR TI=(Risankizumab) OR AB=(Risankizumab) OR TI=(Secukinumab) OR AB=(Secukinumab) OR TI=(Sonelokimab) OR AB=(Sonelokimab) OR TI=(Tildrakizumab) OR AB=(Tildrakizumab) OR TI=(Ustekinumab) OR AB=(Ustekinumab) OR TI=(interleukin-12/23 inhibitor*) OR AB=(interleukin-12/23 inhibitor*) OR TI=(interleukin-17 inhibitor*) OR AB=(interleukin-17 inhibitor*) OR TI=(interleukin-23 inhibitor*) OR AB=(interleukin-23 inhibitor*)  #7 OR #8  #3 AND #6 AND #9 | 737 |
| Embase | ‘Psoriasis’  ‘Psoriases’:ab, ti OR ‘plaque psoriasis’:ab, ti  #1 OR #2  ‘Recurrences’ OR ‘drug tapering’  ‘Recurrences’:ab,ti OR ‘Recrudescence’:ab,ti OR ‘Recrudescences’:ab,ti OR ‘Relapse’:ab,ti OR ‘Relapses’:ab,ti OR ‘interruption’:ab,ti OR ‘stopped’:ab,ti OR ‘stop’:ab,ti OR ‘discontinuation’:ab,ti OR ‘discontinue’:ab,ti OR ‘withdrawn’:ab,ti OR ‘withdrawal’:ab,ti  #4 OR #5  ‘Biological Products’  ‘biologics’:ab,ti OR ‘biologicals’:ab,ti OR ‘biological agent’:ab,ti OR ‘targeted therapies’:ab,ti OR ‘tnf inhibitor*’:ab,ti OR ‘antitumor necrosis factor’:ab,ti OR ‘anti tumour necrosis factor’:ab,ti OR ‘IL-12/23 inhibitor*’:ab,ti OR ‘IL-17 inhibitor*’:ab,ti OR ‘IL-23 inhibitor*’:ab,ti OR ‘Abatacept’:ab,ti OR ‘Adalimumab’:ab,ti OR ‘Bimekizumab’:ab,ti OR ‘Brodalumab’:ab,ti OR ‘Certolizumab’:ab,ti OR ‘Certolizumab Pegol’:ab,ti OR ‘Deucravacitinib’:ab,ti OR ‘Etanercept’:ab,ti OR ‘Golimumab’:ab,ti OR ‘Guselkumab’:ab,ti OR ‘Infliximab’:ab,ti OR ‘Ixekizumab’:ab,ti OR ‘Netakimab’:ab,ti OR ‘Risankizumab’:ab,ti OR ‘Secukinumab’:ab,ti OR ‘Sonelokimab’:ab,ti OR ‘Tildrakizumab’:ab,ti OR ‘Ustekinumab’:ab,ti OR ‘interleukin-12/23 inhibitor*’:ab,ti OR ‘interleukin-17 inhibitor*’:ab,ti OR ‘interleukin-23 inhibitor*’:ab,ti OR  #7 OR #8  #3 AND #6 AND #9  Filter: article or article in press | 1444 |
| Cochrane Library | (psoriasis):ab,ti,kw OR (Psoriases):ab,ti,kw OR (plaque psoriasis):ab,ti,kw  (Recurrence):ab,ti,kw OR (Recurrences):ab,ti,kw OR (Recrudescence):ab,ti,kw OR (Recrudescences):ab,ti,kw OR (Relapse):ab,ti,kw OR (Relapses):ab,ti,kw OR (drug tapering):ab,ti,kw OR (interruption):ab,ti,kw OR (stopped):ab,ti,kw OR (stop):ab,ti,kw OR (discontinuation):ab,ti,kw OR (discontinue):ab,ti,kw OR (withdrawn):ab,ti,kw OR (withdrawal):ab,ti,kw  (Biological Products):ab,ti,kw OR (biologics):ab,ti,kw OR (biologicals):ab,ti,kw OR (biological agent):ab,ti,kw OR (targeted therapies):ab,ti,kw OR (tnf inhibitor*):ab,ti,kw OR (antitumor necrosis factor):ab,ti,kw OR (anti tumour necrosis factor):ab,ti,kw OR (IL-12/23 inhibitor*):ab,ti,kw OR (IL-17 inhibitor*):ab,ti,kw OR (IL-23 inhibitor*):ab,ti,kw OR (Abatacept):ab,ti,kw OR (Adalimumab):ab,ti,kw OR (Bimekizumab):ab,ti,kw OR (Brodalumab):ab,ti,kw OR(Certolizumab):ab,ti,kw OR(Certolizumab Pegol):ab,ti,kw OR(Deucravacitinib):ab,ti,kw OR(Etanercept):ab,ti,kw OR(Golimumab):ab,ti,kw OR(Guselkumab):ab,ti,kw OR(Infliximab):ab,ti,kw OR(Ixekizumab):ab,ti,kw OR(Netakimab):ab,ti,kw OR(Risankizumab):ab,ti,kw OR(Secukinumab):ab,ti,kw OR(Sonelokimab):ab,ti,kw OR(Tildrakizumab):ab,ti,kw OR(Ustekinumab):ab,ti,kw OR(interleukin-12/23 inhibitor*):ab,ti,kw OR(interleukin-17 inhibitor*):ab,ti,kw OR(interleukin-23 inhibitor*):ab,ti,kw  #1 AND #2 AND #3  Filter: clinical trial | 630 |

eTable 2. Effect value extraction strategy

|  | **Effect value extraction strategy** |
| --- | --- |
| Effect values are available in the text or supplementary material. | Prioritize the extraction of results with multiple corrections. |
| Although effect values are not directly available in the text or supplementary material, the original data from which these values were calculated are provided. | The R.4.3.3 software was utilized for statistical analysis; the ‘clogit’ function was employed to calculate odds ratios (OR), while the ‘coxph’ function was used to compute risk ratios (RR) and hazard ratios (HR). |
| The effects of risk factors were presented as mean ± standard deviation (SD) in the text or supplementary material. | $ln(RoM)=ln\left( \frac{{mean}_{exp}}{{mean}_{contr}} \right)$(1)  $SE\left[ ln\left( RoM \right) \right]=\sqrt{\frac{1}{n_{exp}}\left( \frac{{SD}_{exp}}{{mean}_{exp}} \right)^{2}+\frac{1}{n_{contr}}\left( \frac{{SD}_{contr}}{{mean}_{contr}} \right)^{2}}$(1) |
| Survival curves for the risk factors were included in the text or supplementary material. | The Engauge Digitizer software was used to extract the node information from the survival curves, and the extracted data were input into the effect value calculation tool developed by Tierney et al. (2) to obtain HR values. |

**eTable 3. Effect value conversion strategy**

| **HR convert to RR**(3) | **OR convert to RR**(4) |
| --- | --- |
| The formula for HR is similar to the RR value, and in this study, which is a meta-analysis of non-survival data, RR ≈ HR. | If the incidence of the outcome event in the control group is <10% or >90%,  RR ≈ OR;  If the incidence of the outcome event in the control group is between 10 and 90%,  $RR\approx OR\div[(1-P0)+(P0\times OR)]$ |


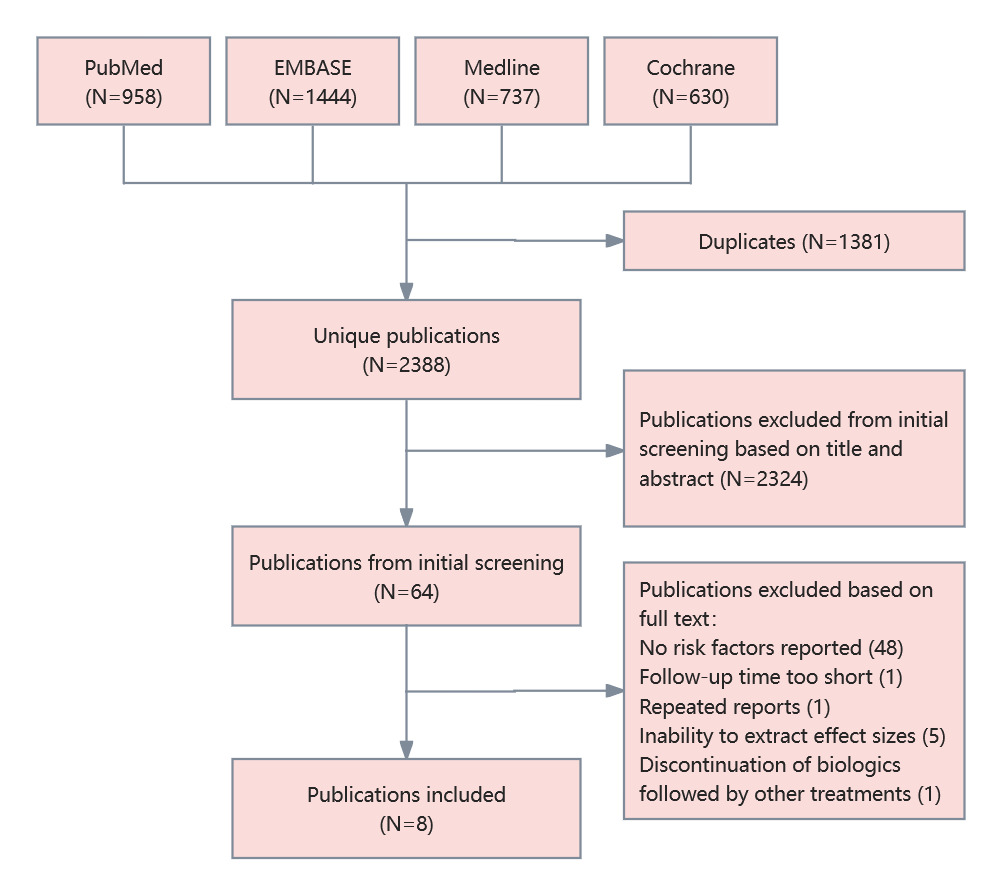


**eFigure 1.** Flowchart of literature search and screening


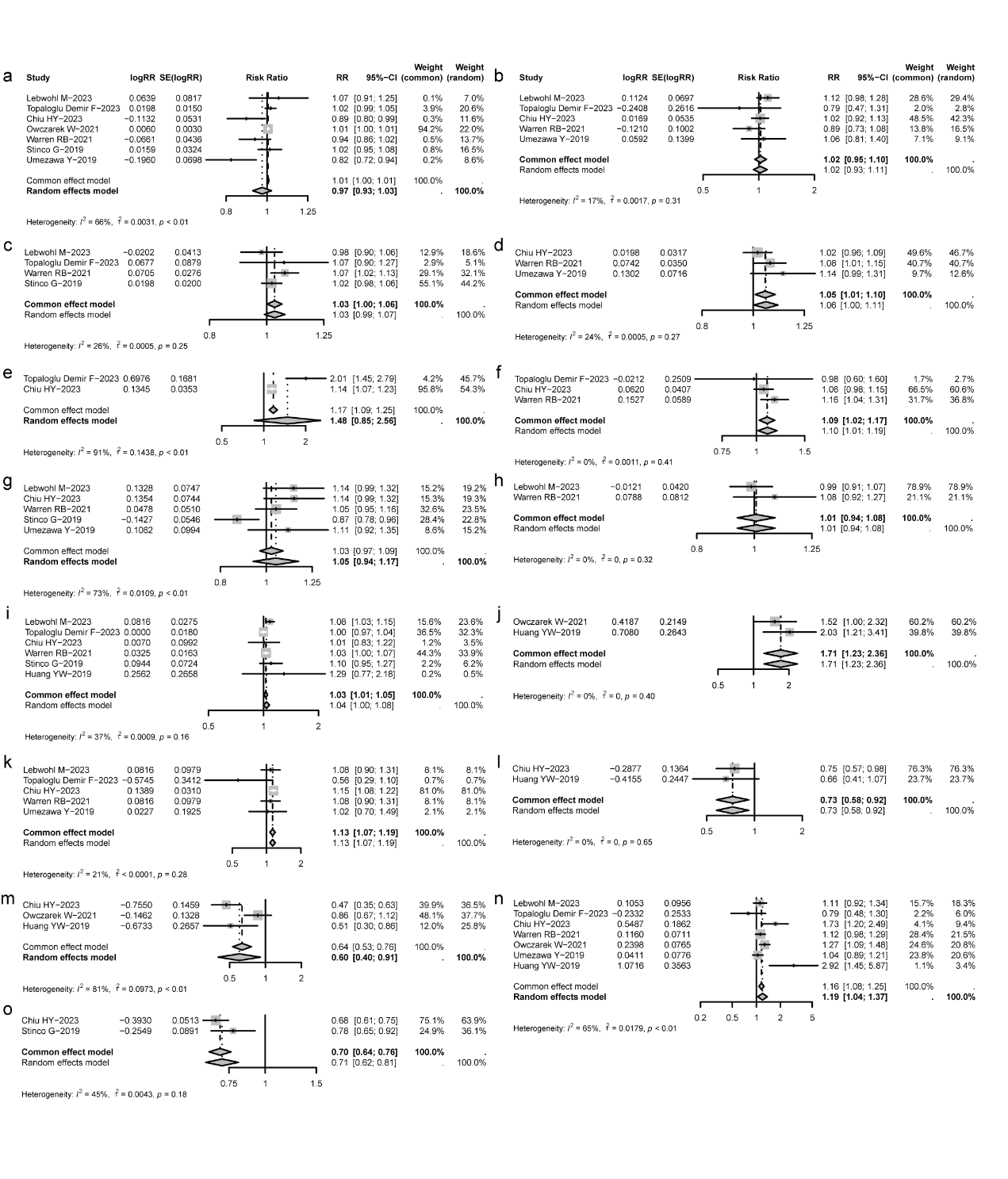


**eFigure 2.** Meta-analysis, heterogeneity test and sensitivity analysis of risk factors for psoriasis relapse after discontinuation of biologics. (a) Age, (b)gender (male), (c) BMI, (d) body weight, (e) drinking, (f) smoking, (g) baseline PASI, (h) baseline BSA, (i) disease course (years), (j) disease course (≥2 years), (k) PsA, (l) faster lesion relief, (m) higher lesion relief, (n) previous exposure to biologics, (o) treatment course

**eTable 4.** Demographic information of the studies included in the meta-analysis

| Study | Lebwohl M. et al (2023) | Topaloğlu Demir F. et al (2023) | Warren R.B. et al (2021) | Owczarek W. et al (2021) | Chiu H.Y. et al (2023) | Stinco G. et al (2019) | Umezawa Y. et al (20190 | Huang Y.W. et al (2019) |
| --- | --- | --- | --- | --- | --- | --- | --- | --- |
| Eligible participants | 220 | 196 | 233 | 705 | 304 | 270 | 70 | 95 |
| Age | 45.81±13.58 | 43.70±14.50 | 45.90±12.34 | 43.80±13.20 | 44.7±13.4 | 51.3±13.7 | 45.1 (11.35) | — |
| Gender (Male) | 144 (65.5%) | 90 (53.3%) | 168 (72.1%) | 467 (66.2%) | 225 (74.0%) | 185 (68.5%) | 56 (80.0%) | — |
| BMI (kg/m^2^) | 28.12±6.03 | 28.10±5.10 | 30.06±7.24 | 84.00±18.90* | 75.20±14.90* | 26.40±4.60 | 73.40±16.43* | — |
| Smoking | — | 62 (36.7%) | 152 (65.2%) | — | 127 (41.8%) | 101 (37.4%) | — | — |
| Drinking | — | 9 (5.3%) | — | — | 64 (21.1%) | 106 (39.3%) | — | — |
| Treatment course (months) | 16.95±12.54 | 19.90±11.50 | 15.41±12.06 | 18.90±11.30 | 14.80±8.20 | 19.95±11.81 | 15.00±9.73 | — |
| PsA | 38 (17.3%) | 57 (33.7%) | 40 (17.2%) | — | 90 (29.6%) | 112 (41.5%) | 10 (14.3%) | — |
| PASI | 22.80±10.07 | 16.50±8.90 | 19.97±8.27 | 23.30±6.70 | 18.6±8.1 | 14.70±7.55 | 26.50±8.75 | — |
| Previous exposure to biologics | 39 (17.7%) | 99 (58.6%) | 47 (20.2%) | 162 (23.0%) | 181 (59.5%) | — | 18 (25.7%) | 26 (27.4%) |

* Body weight instead of BMI

**eFigure 3.** ROC curve analysis of treatment duration to predict psoriasis relapse


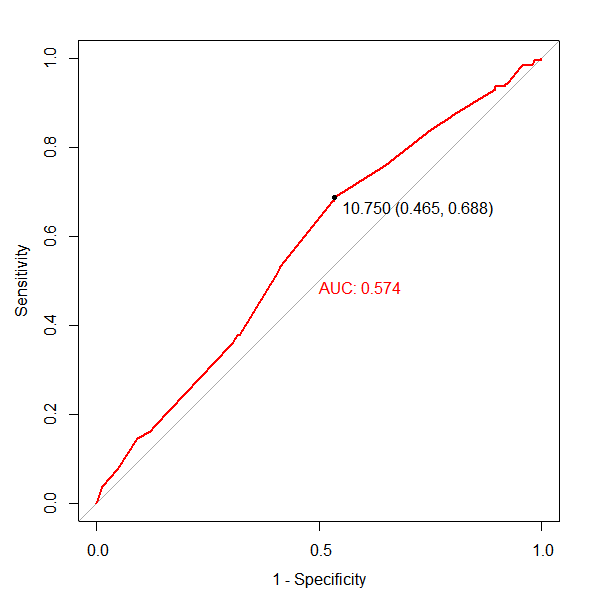


**References**

1. Friedrich JO, Adhikari NK, Beyene J. Ratio of Means for Analyzing Continuous Outcomes in Meta-Analysis Performed as Well as Mean Difference Methods. J Clin Epidemiol (2011) 64(5):556-64. doi: 10.1016/j.jclinepi.2010.09.016.

2. Tierney JF, Stewart LA, Ghersi D, Burdett S, Sydes MR. Practical Methods for Incorporating Summary Time-to-Event Data into Meta-Analysis. Trials (2007) 8:16. Epub 20070607. doi: 10.1186/1745-6215-8-16.

3. Xu T, Zhang YH. Association of psoriasis with stroke and myocardial infarction: meta-analysis of cohort studies. *Br J Dermatol.* Dec 2012;167(6):1345-50. doi:10.1111/bjd.12002

4. Viera AJ. Odds ratios and risk ratios: what's the difference and why does it matter? *South Med J.* Jul 2008;101(7):730-4. doi:10.1097/SMJ.0b013e31817a7ee4
